# Emergence of *Fusarium incarnatum* and *Fusarium avenaceum* in wilt affected solanaceous crops of the Northern Himalayas

**DOI:** 10.1038/s41598-025-87668-3

**Published:** 2025-01-31

**Authors:** Tasmeen J. Parihar, Madeeha Naik, Shafqat Mehraj, Syed Inam ul Haq, Maqsooda Perveen, Ishfaq Ahmed Malla, Taniya Abid, Nadia Gul, Khalid Z. Masoodi

**Affiliations:** 1 Transcriptomics Laboratory (K-Lab), Division of Plant Biotechnology, Sher-e-Kashmir University of Agricultural Sciences and Technology of Kashmir, Shalimar, Srinagar, Jammu and Kashmir 190025 India; 2 College of Temperate Sericulture, Sher-e-Kashmir University of Agricultural Sciences and Technology of Kashmir, Shalimar, Srinagar, Jammu and Kashmir 190025 India

**Keywords:** *Fusarium* wilt, Solanaceous crops, Molecular techniques, *Fusarium incarnatum*, *Fusarium avenaceum*, Crop pathogens, Biotechnology, Microbiology

## Abstract

The objective of this study was to identify and characterize the fungal pathogens responsible for wilt diseases in solanaceous crops, specifically tomato, brinjal, and chili, in the Kashmir valley. Through both morphological and molecular analyses, including DNA barcoding of the ITS, TEF, RPB1, and RPB2 genomic regions, *Fusarium incarnatum* and *Fusarium avenaceum* were identified as the primary causal agents of wilt in tomato and brinjal, and chili, respectively. Pathogenicity tests confirmed the virulence of these pathogens, with typical wilt symptoms observed upon inoculation. This represents the first report of *F. incarnatum* and *F. avenaceum* as wilt pathogens in solanaceous crops in India. Phylogenetic analysis further confirmed the genetic variability of these pathogens, revealing their expanding host range. The findings underscore the growing adaptability of these *Fusarium* species to diverse agricultural systems and highlight the urgent need for targeted disease management strategies to mitigate the significant yield losses caused by *Fusarium* wilt in solanaceous vegetable production.

## Introduction

The family Solanaceae comprises several economically important flowering plants, including crops that serve various purposes such as food, medicine, and ornamentals. Among the most significant solanaceous vegetables are tomato, eggplant, and pepper, which are known for their numerous health benefits^1^. However, these crops are highly susceptible to a range of diseases, with fungal wilt being one of the most destructive, both in terms of incidence and yield loss^2^. In India, wilt disease in solanaceous crops has emerged as a severe threat, with disease incidence ranging from 5 to 93%, and yield losses between 45 and 60% due to the invasion of *Fusarium pallidoroseum* in the Kashmir valley^3^. Notably, *Fusarium solani sp. melongenae* has been confirmed as the causal agent of wilt in brinjal in Jammu and Kashmir^4^, and several other *Fusarium* species, including *Fusarium chlamydosporum, Fusarium equiseti*, and *Fusarium flocciferum*, have been recently reported as pathogens in chili and brinjal in the Kashmir region^5,6^. The occurrence of wilt disease in the Kashmir valley has been frequent, leading to yield losses ranging from 11.67 to 96.67%^7^. *Fusarium* wilt has thus become a major concern in solanaceous crops, especially as the pathogens responsible have diversified across different host species. The current study focuses on the incursion of these wilt-causing pathogens in the Kashmir valley, particularly the movement of *Fusarium* species between various host crops. Traditional identification of *Fusarium* pathogens based on morphological features, such as macroconidia, microconidia, and chlamydospores, has been insufficient due to the complex nature of the Fusarium genus^8,9^. Thus, DNA sequence-based identification has become an invaluable tool for accurately identifying *Fusarium* species. Molecular techniques, such as DNA barcoding using the internal transcribed spacer (ITS) and translation elongation factor (TEF) genomic regions, as well as the RPB1 and RPB2 genes, have provided clearer insights into the diversity of these pathogens^8,10^. Previous studies have used ITS as a DNA barcoding marker to identify *Fusarium* species, but it has been noted that ITS may not be reliable for closely related species^11^. Consequently, conserved genes like TEF have been increasingly employed for phylogenetic analyses in combination with ITS to better resolve species relationships^12^. For instance, the TEF gene has proven effective in distinguishing subspecies and has been used by several researchers to clarify species identities within the *Fusarium* genus^11,13,14^. In this investigation, we identified *Fusarium avenaceum* as the predominant wilt-causing pathogen in chili and *Fusarium incarnatum* as the primary pathogen in brinjal and tomato. This finding represents a new report of *F. incarnatum* and *F. avenaceum* as causal agents of wilt in solanaceous crops in India. The increasing rate of pathogen invasions, exacerbated by changes in agricultural practices, habitat disturbances, climate change, and pathogen introduction through trade, further complicates the management of these diseases. This study contributes to the understanding of *Fusarium* species’ geographical spread and their potential for cross-host infection in solanaceous crops.

## Material and methods

### Sample collection and maintenance of cultures

Diseased plants of chili, tomato, and brinjal, showing typical symptoms of wilt disease, were collected from different districts of the Kashmir region, including Pulwama, Srinagar, Baramulla, and Anantnag, during July and August 2020. Samples were randomly selected from various districts of the Kashmir valley. The tissue bit technique was used to isolate the fungus from the infected samples, which were then purified using the single-spore technique. The pure cultures obtained were maintained at 25 °C ± 1 °C and stored at 4 °C^15^.

### Morphological and cultural characteristics of the isolated pathogen(s)

The pathogens were cultured on Potato Dextrose Agar (PDA) medium at 25 °C to obtain pure cultures, which were then subjected to morphological studies. Cultures that were 10–15 days old were used to prepare semi-permanent slides for microscopic observation. The key characteristics examined included the shape, size, and septation of microconidia, macroconidia, and mycelium.

### Identification and pathogenicity test

The pathogens were identified based on their pathological and morphological characteristics. Seedlings of chili (cv. Kashmir Long-1), tomato (cv. Shalimar Hybrid Tomato-1), and brinjal (cv. Local Long) were uprooted and transplanted into the infected potting mixture. The plants were continuously monitored for symptom development until they eventually died, thereby fulfilling Koch’s postulates^15,16^.

### DNA extraction

For DNA extraction, mycelia were crushed in 400 µl of extraction buffer using a mortar and pestle, and the resulting slurry was incubated at 65 °C for 1 h. After the addition of RNase and a subsequent incubation, sodium acetate was introduced, and the sample was chilled. The lysate was then centrifuged, and DNA was precipitated with isopropanol, pelleted, washed with 70% ethanol, air-dried, and resuspended in Tris–EDTA buffer.

### PCR amplification

For PCR amplification, a 25 μl reaction mixture was prepared, with an initial denaturation at 95 °C for 8 min, followed by 35 cycles of denaturation at 95 °C for 15 s, annealing based on primer Tm, first extension at 72 °C for 1 min and a final extension at 72 °C for 25 min. PCR products were run on a 1% agarose gel with SYBER green dye in 1 × TAE buffer, visualized with a gel documentation system, and compared to a 100 bp DNA ladder. Amplified products from ITS, TEF, RPB1, (Tables 1, 2 and 3) and RPB2 primers were sequenced by Bionivid Technology for DNA barcoding to analyze genetic variability.

**Table 1:** Primers along with sequences and Tm used for amplification of ITS regions.

S. no	Primer name	Primer sequence	Tm °C
1	K-Lab-FusOxy-ITS1F2 Primer	5’CCTGCGGAGGATCATTA 3’	63.7
2	K-Lab-FusOxy-ITS4R2 Primer	5’TCCTCCGCTTATTGAT3’	53.6

**Table 2:** Primers along with sequences and Tm used for amplification of TEF region.

S. no	Primer name	Primer sequence	Tm °C
1	K-Lab-TEF-Fu3-F	5’GGTATCGACAAGCGAACCAT3’	63.8
2	K-Lab-TEF-Fu3-R	5’TAGTAGCGGGAGTCTCGAA3	63.8

**Table 3:** Primers along with sequences and Tm used for amplification of RPB1 and RPB2 gene.

S. no	Primer name	Primer sequence	Tm °C
1	K-Lab-RPB1 -Fa -F	5’CAYAARGARTCYATGATGGGWC3’	55
2	K-Lab-RPB1-G2R -R	5’GTCATYTGDGTDGCDGGYTCDCC3’	55
3	K-Lab-RPB2-5F2 -F	5’GGGGWGAYCAGAAGAAGGC3’	55
4	K-Lab-RPB2-7CR -R	5’CCCATRGCTTGYTTRCCCAT3’	55

### Sequencing and DNA barcoding

Amplified PCR products using ITS1F2 / ITS4R2 and TEF Fu3 F/ TEF Fu3, RPB1 and RPB2 were outsourced to Bionivid Technology [P] Limited, Bangalore. The genetic variability among collective samples was ascertained through DNA barcoding of ITS, TEF, RPB1 and RPB2 regions. A dendrogram was constructed using Cluster W software.

## Results

**Fig. 1:**
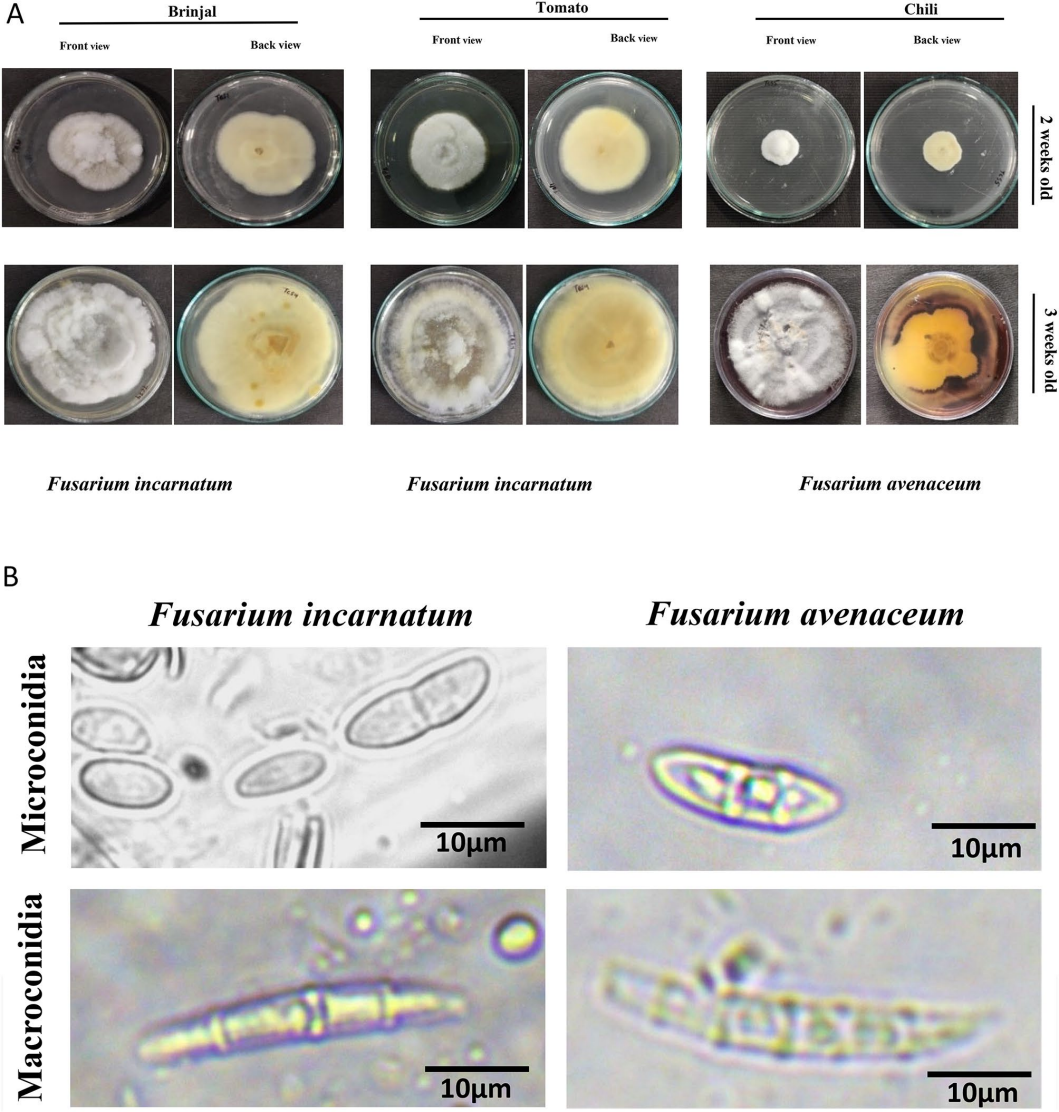
(**a**) Cultural characterisation of *Fusarium incarnatum* and *Fusarium avenaceum* infecting tomato, brinjal and chili. (**b**) Morphological characteristics of *Fusarium incarnatum* and *Fusarium avenaceum* causing wilt solanaceous host crops.

Tomato and brinjal plants showing wilt symptoms were collected from various regions of Kashmir, including Anantnag, Srinagar, Pulwama, and Baramulla, during July and August 2020. The observed symptoms included yellowing of the plant, lesions on the roots and stems, and wilting or death of the plant. The collected samples were isolated on PDA medium, and pure cultures were maintained at 25 ± 1 °C and stored at 4 °C (Fig. 1a).

### Morpho-cultural identification

To identify the pathogen on the host, pure cultures were established on PDA medium, and both were analyzed based on their morphological characteristics. The key features studied included the shape, size, and septation of microconidia, macroconidia, and mycelium (Table 4). *Fusarium incarnatum*, isolated from tomato and brinjal, initially produced white colonies that gradually turned yellow at the agar base, with a growth of 90 mm after 18 days of incubation at 25 ± 1 °C. Microscopic observations revealed that the mycelium was branched and cylindrical, with a width of 3.20–4.20 µm. Microconidia were single-celled, hyaline, with 0–1 septa, and measured 10–12 × 3–4 µm. Macroconidia were sickle-shaped, hyaline, 4–5 septate, and measured 28–31 × 3–5 µm. *Fusarium avenaceum*, isolated from the chili host, initially produced white colonies that gradually turned brownish-yellow at the agar base. The fungal culture reached a growth of 90 mm after 18 days of incubation at 25 ± 1 °C. The average measurements indicated that the mycelium was branched and cylindrical, measuring 53–4.98 µm in diameter. Microconidia were curved, hyaline, with 0–2 septa, and measured 11–20.2 µm × 4–5 µm. Macroconidia were curved, hyaline, with 5–7 septa, and measured 49.9–78 × 3.6–4.7 µm (Fig. 1b).

**Table 4:** Morphological characteristics of the *Fusarium* infecting chili, tomato and brinjal collected from different regions of Kashmir valley.

S. no	Isolate name	Mycelium	Microconidia			Macroconidia		
			Shape	Size (µm)	Septation	Shape	Size (µm)	Septation
1	*Fusarium incarnatum*	Branched, cylindrical mycelium	Single celled	10–12 × 3–4 µm	0–1 septate	Sickle shaped	28–31 × 3–5 µm	4–5 Septate
2	*Fusarium avenaceum*	Cylindrical	Curved	11–20.2 × 4–5	1–2 Septate	Curved	49.9–78 × 3.6–4.7	5–7 Septate

### Pathogenicity test

**Fig. 2:**
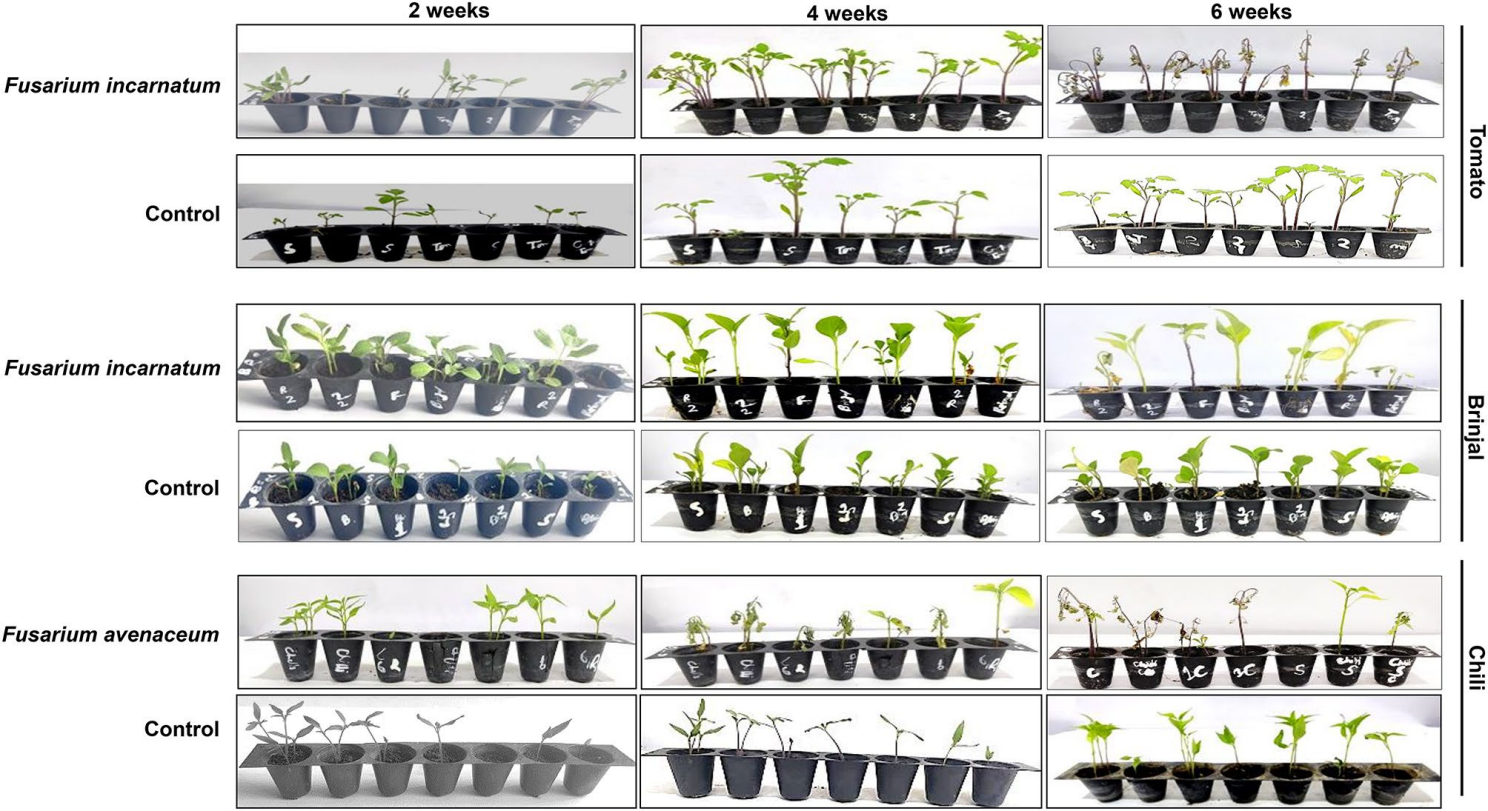
Pathogenicity test of different *Fusarium incarnatum and Fusarium avenaceum* species with respect to tomato, brinjal and chili.

The seedlings of chili (cv. Kashmir Long-1), tomato (cv. Shalimar Hybrid Tomato-1), and brinjal (cv. Local Long) were potted in the greenhouse. *Fusarium incarnatum*, obtained from the respective crop plant, was used to infect the same potted plant to fulfill Koch’s postulates^17,18^. The incubation period for *Fusarium incarnatum* was recorded as four weeks for symptom development, while *F. avenaceum* took six weeks for symptom appearance (Fig. 2). Infected plants exhibited initial symptoms, with leaves discoloring from light green to yellow, followed by drooping, shriveling, and eventually death. The collar region of the plants was cut vertically, revealing brownish spots and discoloration in the vascular bundles, indicating wilt as the cause of death. The pathogens were re-isolated and inoculated from all infected plants. These were compared with the original pure culture inoculates, and the re-isolates resembled the original cultures based on morphological, cultural, and pathogenic characteristics.

### DNA Extraction PCR amplification

**Fig. 3:**
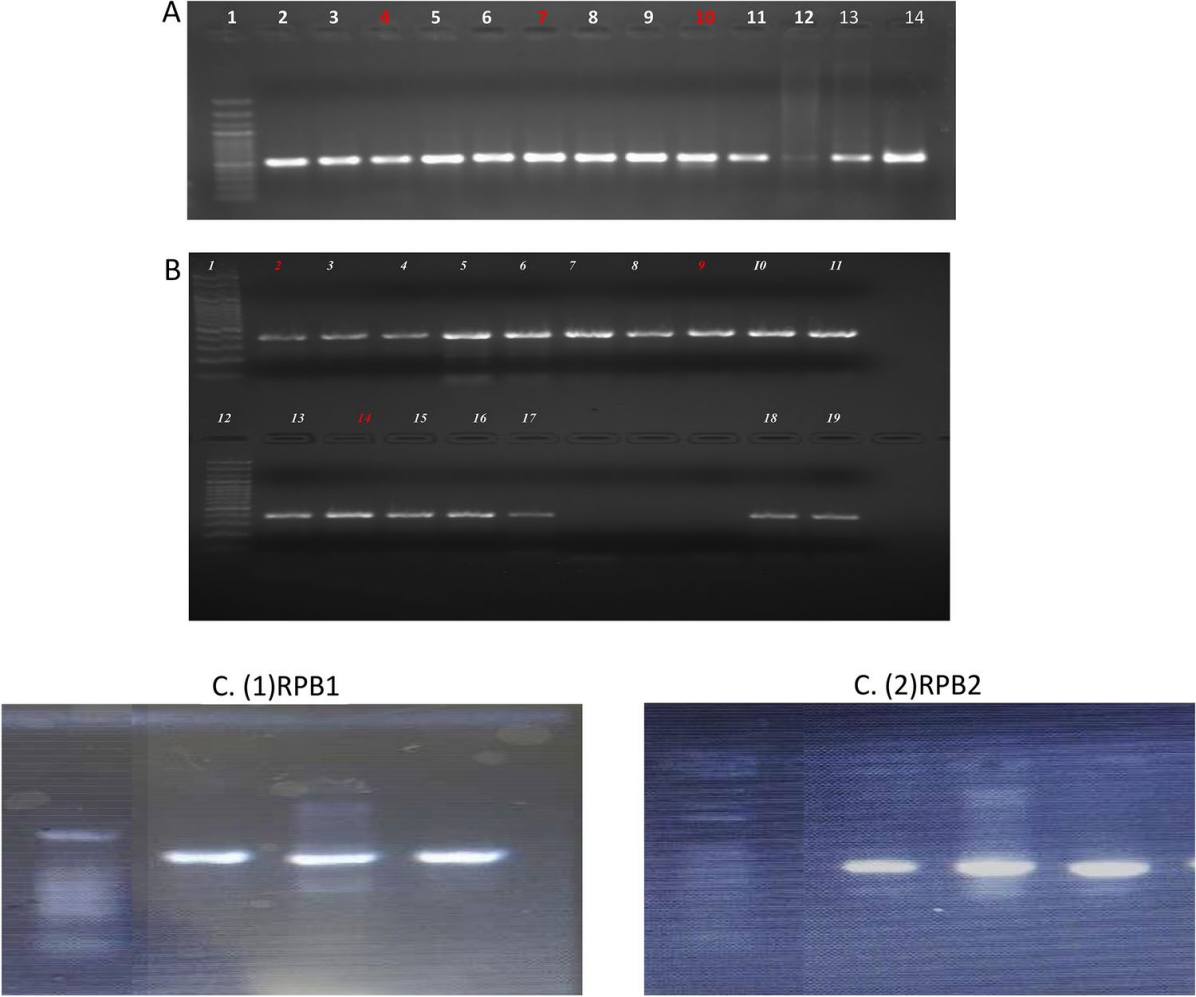
(**a**) PCR amplification of isolates using ITS ( Lane 4, 7 and 9). (**b**) PCR amplification of isolates using TEF ( Lane 2, 9 and 14). (**c**) PCR amplification of isolates using RPB1 and RPB2 genes. M is 100 bp ladder.

DNA extraction of isolates followed by PCR amplification was performed for ITS1 and ITS4, TEF, RPB1 and RPB2 primer pairs, and the amplified products were run on 1% agarose gel (Fig. 3a–c). Amplified PCR products were outsourced for sequencing to Bionivid Technology [P] Limited, Bangalore. BLAST was used to find regions of similarity between the query sequence and the sequence present in the NCBI (www.ncbi.nlm.in) database. The sequences were successfully submitted and Accessioned in GenBank (Table 5). *Fusarium incarnatum* and *Fusarium avenaceum* have been first time reported as wilt pathogens of solanaceous host crops (Table 6).

**Table 5:** Accession numbers of *Fusarium incarnatum* and *Fusarium avenaceum* in NCBI Genbank collected from different districts of Kashmir valley.

S. no	Place of collection	Pathogen identified	Host plant	Accession number (ITS)	Accession number (TEF)	Accession number (RPB1)	Accession number (RPB2)
1	Pulwama (koel)	*Fusarium incarnatum*	Brinjal	OM189449	OM441190	OR484033	OR484035
2	Srinagar (Shalimar)	*Fusarium incarnatum*	Tomato	OM189460	OM441201	OR484034	OR484036
3	Anantnag (Kokarnag)	*Fusarium avenaceum*	Chili	OM189456	OM441197	OR640336	OR640337

**Table 6:** Diversification of *Fusarium incarnatum* previously reported and new reports with respect to host crops.

Species	Initially reported in (Host plant)	New report (Host plant)
*Fusarium incarnatum*	Sorghum (*Sorghum bicolor*)	Brinjal (*Solanum melongena*)
	Rice (*Oryza sativa*)	
	Maize (*Zea mays*)^21^	
	Bell pepper (*Capsicum annuum* L.)^20^	Tomato (*Solanum lycopersicum*)
*Fusarium avenaceum*	Wheat (*Triticum*)^22^	Chili (*Capsicum annuum*)
	Soybean (*Glycine max*)^23^	

### Phylogenetic analysis

**Fig. 4:**
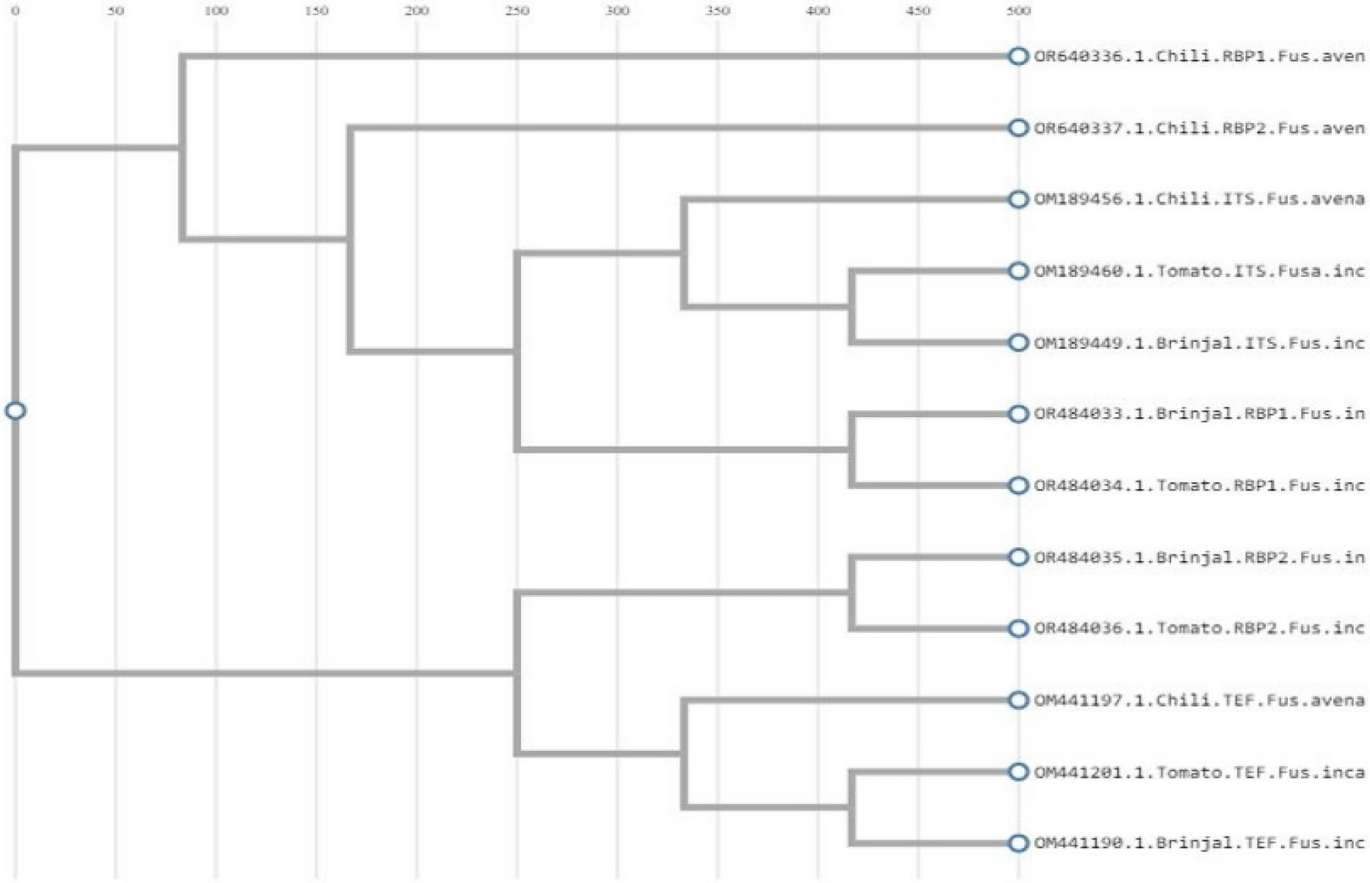
Phylogenetic relationship of *Fusarium incarnatum* and *Fusarium avenaceum* causing wilt in solanaceous crops using Cluster W.

A dendrogram was generated using Cluster W software. The phylogenetic analysis using ITS, TEF and RPB1, RPB2 sequences were carried out (Fig. 4).

## Discussion

The major solanaceous vegetable crops include tomato, eggplant, chili, and pepper^1^. These crops offer numerous health benefits and help combat various diseases. However, the most devastating disease, which is becoming increasingly widespread, is wilt, particularly in terms of incidence and yield loss. *Fusarium* wilt leads to 50–80% yield losses annually^8^. In the present study, fungi associated with wilt in tomato and brinjal were identified as *Fusarium incarnatum*, while *Fusarium avenaceum* was identified as the pathogen in chili, based on both morphological and molecular characteristics of the pathogens^19^. When artificially inoculated using the single-spore technique on the respective hosts, with rhizosphere inoculation on wilted plants, the fungal pathogens isolated from wilted plants exhibited typical disease symptoms. The plants developed initial symptoms by the second week of inoculation. The plants showed complete wilting, beginning with light green to yellowish discoloration of the leaves, followed by drooping, shriveling, and, finally, death of the entire plant by the sixth week of inoculation. Upon vertical cutting of the collar region, the vascular bundles showed brownish discoloration.

Amplification of the internal transcribed spacer (ITS) region using genus- and species-specific ITS primers identified *Fusarium incarnatum* and *Fusarium avenaceum* using primer combinations such as K-Lab-FusOxy-ITS1F2 and Lab-FusOxy-ITS4R2. To further confirm these results, transcription elongation factor (TEF Fu3) amplification was performed with the TEF primer combination (TEF-Fu3-F and TEF-Fu3-R), as well as RPB1 and RPB2 genes. The PCR products of the ITS1-5.8S-ITS4 region, TEF Fu3, and RPB1 and RPB2 genes were sequenced, and the pathogens identified through sequencing were published in GenBank. Sequence alignment using Cluster W revealed that the similarity level among sequences was independent of their geographical origin^20,21^. Furthermore, *Fusarium incarnatum* has previously been reported in crops such as sorghum, rice, maize, and bell pepper, while *Fusarium avenaceum* has been identified in wheat and soybean^22,23^. In solanaceous crops, these species have been identified as wilt pathogens for the first time in India and the World, highlighting the diversifying nature of *F. incarnatum* and *F. avenaceum* with respect to their host crops, both within solanaceous crops and other host crops. In the phylogenetic study, the ITS, TEF, RPB1, and RPB2 genes were grouped into four distinct clusters. Sequences were compared with their respective hits retrieved from the NCBI database.

## Conclusion

This study provides significant insights into the fungal pathogens causing wilt in major solanaceous crops, identifying *Fusarium incarnatum* and *Fusarium avenaceum* as the causal agents in tomato, brinjal, and chili, respectively. These findings were confirmed through morphological analysis, molecular characterization using ITS, TEF, RPB1, and RPB2 gene markers, and pathogenicity testing via artificial inoculation. The phylogenetic analysis and sequencing revealed that these pathogens, though previously reported in other crops like sorghum, rice, maize, wheat, and soybean, are reported for the first time in solanaceous crops in India. This highlights the expanding host range of *F. incarnatum* and *F. avenaceum*, suggesting their evolving adaptability to diverse agricultural systems. The study emphasizes the need for ongoing research and monitoring of *Fusarium* species to understand their host specificity, geographical distribution, and impact on solanaceous crops. Such efforts are crucial for developing targeted management strategies to mitigate the devastating yield losses caused by *Fusarium* wilt in solanaceous vegetable production.

## Data Availability

The sequencing data is available on the NCBI database. The sequence of ITS with accession numbers OM189449, OM189460, OM189456 were successfully deposited in GenBank (www.ncbi.nlm.in). The sequence of TEF has been provided Accession Numbers OM441190, OM441201, and OM441197 were successfully deposited in GenBank www.ncbi.nlm.in. The sequence of RPB1 and RPB2 with accession numbers OR484033, OR484034, OR484035, OR484036, OR640336 and OR640337 were successfully deposited in GenBank www.ncbi.nlm.in. The Fungal material was formally identified by Dr. Zahoor Bhat Plant Pathologist and Dr. Khalid Z. Masoodi Plant Biotechnologist. No voucher specimen is required to be deposited in a publicly accessible herbarium for this study.
